# Recent Advances in the Synthesis of Cyclic Sulfone Compounds with Potential Biological Activity

**DOI:** 10.3390/molecules29245868

**Published:** 2024-12-12

**Authors:** Hong-Li Huang, Ya-Qian Shi, Jia-Xin Ning, Shan Li, Dian-Tao Song, Fei Gao, Na Ma

**Affiliations:** 1 College of Chemistry and Chemical Engineering, Liaocheng University, Liaocheng 252059, China; 2 Shanghai Kinlita Chemical Co., Ltd., Shanghai 201417, China; flyhightly@shu.edu.cn

**Keywords:** cyclic sulfone, catalytic approaches, heterocyclic compounds

## Abstract

In recent years, cyclic sulfone compounds, a subset of biologically active heterocyclic compounds, have gained considerable attention due to their potential in the development of novel active pharmaceutical ingredients. This review focuses on identifying simple, mild, environmentally friendly, and efficient synthesis methods. Various catalytic approaches for the synthesis of cyclic sulfone compounds are systematically reviewed, highlighting their advantages and potential applications in pharmaceutical development.

## 1. Introduction

**Figure 1:**
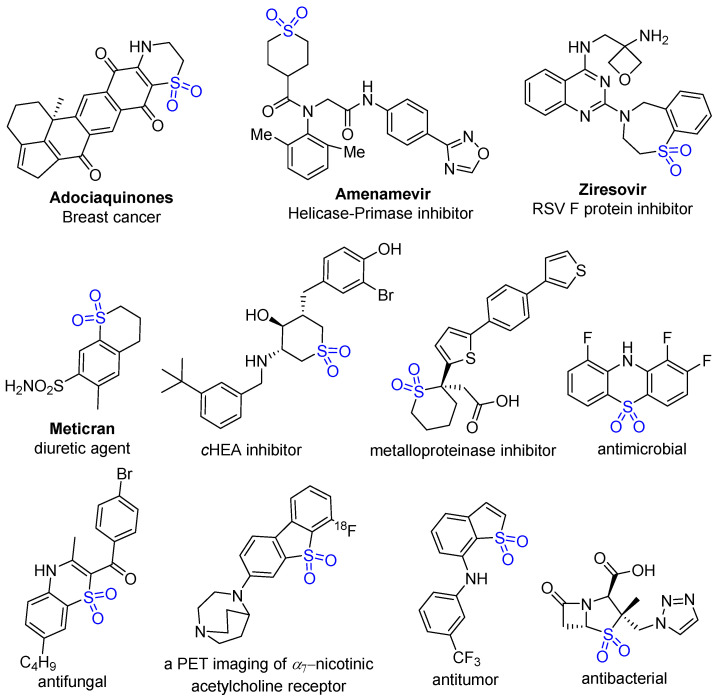
Representative biologically active cyclic sulfone derivatives.

Cyclic sulfones, as a class of heterocyclic compounds, have been widely exploited in organic synthesis, the pharmaceutical industry, and optoelectronic materials due to their remarkable chemical properties [1], biological activities [2], and optoelectronic properties [3]. Several bioactive cyclic sulfones are illustrated in Figure 1. For example, adociaquinones show inhibitory effects on cell proliferation and hold promise for treating diabetes and neurodegenerative diseases [4]. Amenamevir serves as a helicase–primase inhibitor [5], while Ziresovir is a promising respiratory syncytial virus (RSV) protein inhibitor [6]. Meticran functions as a diuretic agent [7]. Moreover, these cyclic sulfones also exhibit inhibitory effects (e.g., acting as a brain-penetrating cyclic hydroxyethylamine (*c*HEA) BACE1 inhibitor, a matrix metalloproteinase inhibitor, and a PET tracer for the imaging of *α*_7_ nicotinic acetylcholine receptors (*α*_7_ nAChRs)) [8,9,10], and demonstrate antimicrobial [11], antifungal [12], and anticancer activities [13] (Figure 1).

**Scheme 1:**
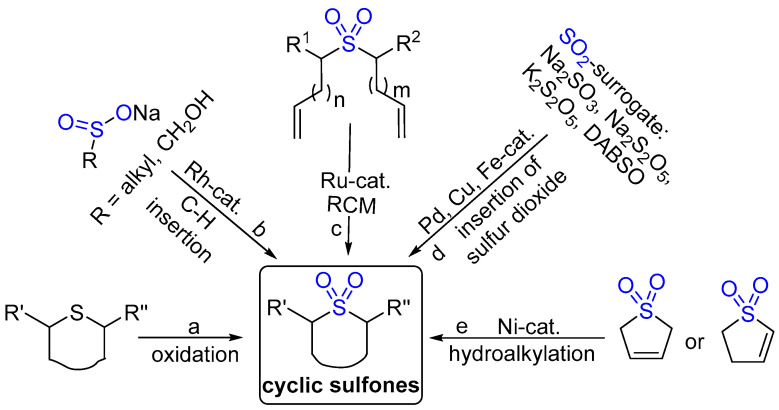
Strategies for the synthesis of cyclic sulfones.

As such, the synthesis of cyclic sulfone frameworks has garnered significant interest from synthetic and pharmaceutical chemists, leading to the development of a variety of efficient approaches [14,15]. In this regard, as depicted in Scheme 1, the current synthetic strategies mainly involve (a) the oxidation of thioethers employing *m*-CPBA, H_2_O_2_, and KMnO_4_ as oxidants [16,17,18], (b) Rh/phase transfer catalyst-catalyzed C-H insertion reaction with sulfonates [19,20], (c) Ru-catalyzed ring-closing metathesis (RCM) reactions of the corresponding dienes [21], (d) transition metal (Pd, Cu, and Fe)/photo-catalyzed insertion of sulfur dioxide (SO_2_) using SO_2_ surrogates [22,23], (e) Ni-catalyzed hydroalkylation reactions [24], and so on [25,26].

Despite recent extensive progress in the synthesis of cyclic sulfones, a timely and comprehensive summary of these advances is still needed. To highlight the key methods for the construction of cyclic sulfones over the last 20 years, this review focuses on various catalytic techniques, including metal-catalyzed cyclization, photocatalytic cyclization, and other emerging methodologies, organized into three sections. These strategies provide valuable insights into the efficient and environmentally friendly synthesis of cyclic sulfone derivatives.

## 2. Metal-Catalyzed Synthesis of Cyclic Sulfone Compounds

**Scheme 2:**
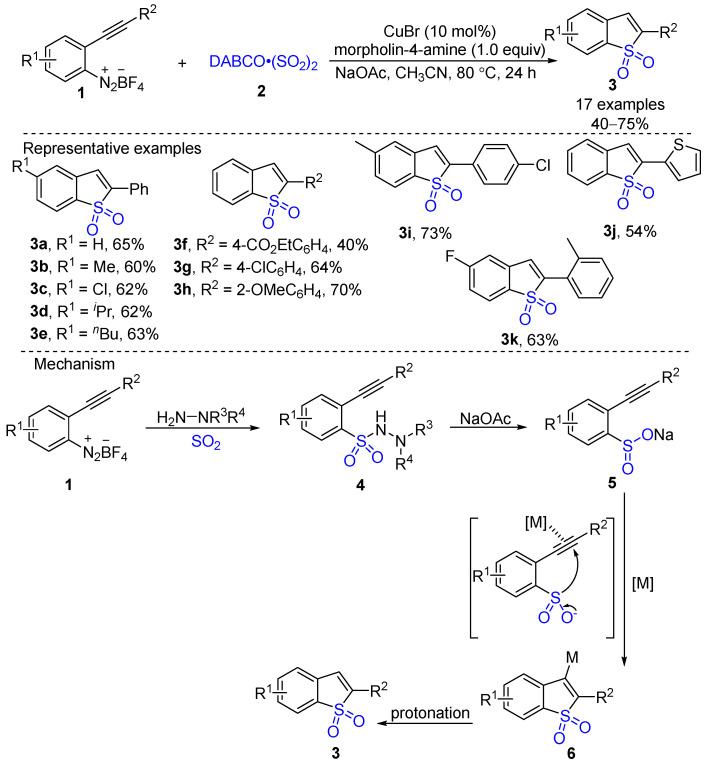
Copper (I)-catalyzed synthesis of benzo*[b]*thiophene 1,1-dioxides.

Recently, the use of SO_2_ as a sulfonyl source for the synthesis of organosulfones has proven to be a powerful strategy, directly introducing the SO_2_ group into organic frameworks [27,28]. Bench-stable solid DABCO-bis(sulfurdioxide) (DABSO) [29], inorganic sulfites M_2_S_2_O_5_ (M = Na, K), and Na_2_SO_3_ are commonly selected as SO_2_ surrogates. Specifically, in 2015, using DABSO as the SO_2_ source, a research team led by Wu developed a copper(I)-catalyzed reaction that transforms 2-alkylalkynyl difluoroborates **1** into benzo*[b]*thiolan-1,1-dioxides **3**, achieving yields of 40–75% (Scheme 2) [30]. This method effectively forms two new C-S bonds through a sequential tandem process.

Under the standard conditions, the reaction exhibited broad applicability and high efficiency. Substrates bearing various functional groups (e.g., F, Cl, Me, and *^n^*Bu) on the aromatic ring performed well in this transformation and successfully yielded the desired products **3**. Additionally, substrates with different aryl (such as 4-ClC_6_H_4_, 4-CO_2_EtC_6_H_4_, 2-OMeC_6_H_4_, and thienyl)-substituted acetylene motifs were tolerated, all proceeding in moderate to good yields. It is noteworthy that the reaction did not occur without morpholin-4-amine, highlighting that the reaction progressed via the N-morpholino-2-(phenylethynyl)benzenesulfonamide intermediate **4**.

Based on the experimental findings, a plausible reaction mechanism was proposed. Initially, 2-alkynylphenyl diazotetrafluoroborate **1** reacts with SO_2_ and morpholin-4-amine, generating intermediate **4**. Subsequently, in the presence of a base, the S-N bond of sodium sulfite is cleaved, forming N-aminosulfonamide **5**. This is followed by a metal-catalyzed intramolecular *5-endo* ring cyclization, which leads to protonation and the formation of the desired product **3** (Scheme 2).

**Scheme 3:**
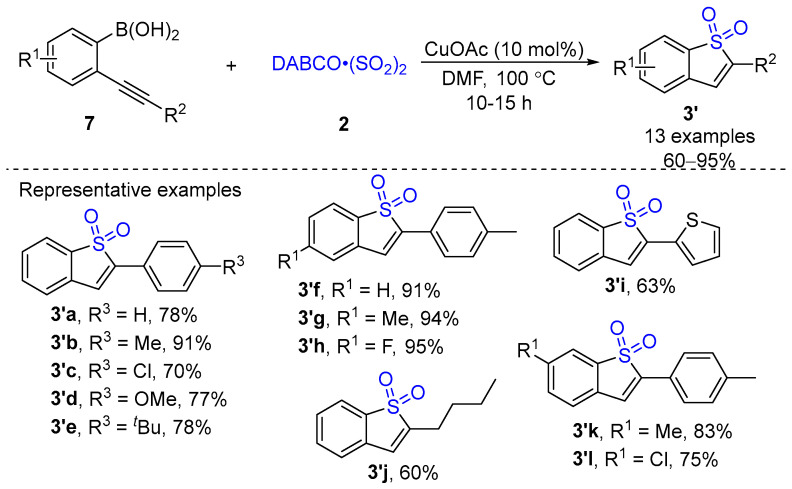
Copper (I)-mediated synthesis of benzo[*b*]thiophene-1,1-dioxides.

Afterwards, in 2016, the same group reported a similar Cu(I)-catalyzed method for the insertion of SO_2_ into (2-alkynyl)boronic acid **7**, resulting in the efficient construction of benzo*[b]*thiolan-1,1-dioxides **3′** in 60–95% yields (Scheme 3) [31]. The reaction was investigated using (2-alkynyl)boronic acid **7** as the model substrate in the presence of 10 mol% of copper (I) acetate in DMF at 100 °C. Under standard conditions, the substrates containing the methyl group and halogens (e.g., F, Cl) on the aromatic ring demonstrated good reactivity, affording the corresponding target products in 75–95% yields. In addition, substrates with various aryl (e.g., 4-ClC_6_H_4_, 4-MeC_6_H_4_, 4-OMeC_6_H_4_, and 4-*^t^*BuC_6_H_4_) substituents (R^2^) attached to the triple bond were explored, affording the expected products in moderate to excellent yields. The *n*-butyl substituted acetylene unit was also screened, successfully obtaining products in 60% yield.

**Scheme 4:**
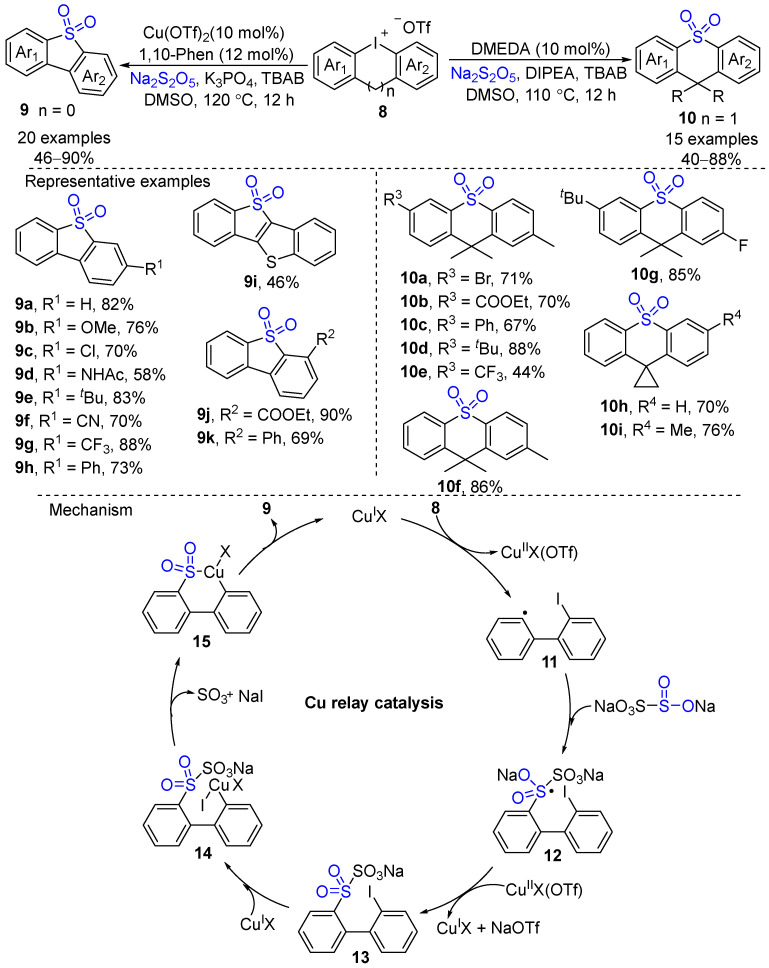
Catalyzed synthesis of functionalized diaryl-annulated sulfones.

Building on this foundation, researchers continued to explore synthetic methods for cyclic sulfones to further expand their application scope. In 2017, Jiang’s research group disclosed a novel synthetic route for constructing diaryl-annulated sulfones molecules **9** and **10** through the SO_2_/iodine (I) exchange of diaryliodonium salts **8** (Scheme 4) [32]. In this process, commercially available inorganic Na_2_S_2_O_5_ served as a safe and convenient SO_2_ surrogate. This synthetic reaction revealed broad functional group compatibility, with a range of functionalized, five-membered **9** and six-membered **10** cyclic-conjugated diaryl-annulated sulfones successfully obtained in moderate to good yields, regardless of whether the benzene ring contained electron-withdrawing (Cl, CN, CF_3_, and COOEt) or electron-donating (Me, OMe, and NHAc) groups.

The proposed mechanism for this reaction involves the formation of a free radical intermediate **11** through the oxidation of Cu(I) to Cu(II). Intermediate **11** then captures Na_2_S_2_O_5_, generating a free SO2 radical anion intermediate **12**. Intermediate **12** is subsequently oxidized to intermediate **13**, with Cu(II) being reduced back to Cu(I). The oxidative addition of aryl iodide to Cu(I) forms the Cu(III) aryl species **14**, which undergoes an intramolecular ligand exchange to yield intermediate **15**. Finally, reduction and elimination of intermediate **15** result in the formation of the desired cyclic sulfone product **9**, along with the regeneration of the Cu(I) catalyst (Scheme 4).

**Scheme 5:**
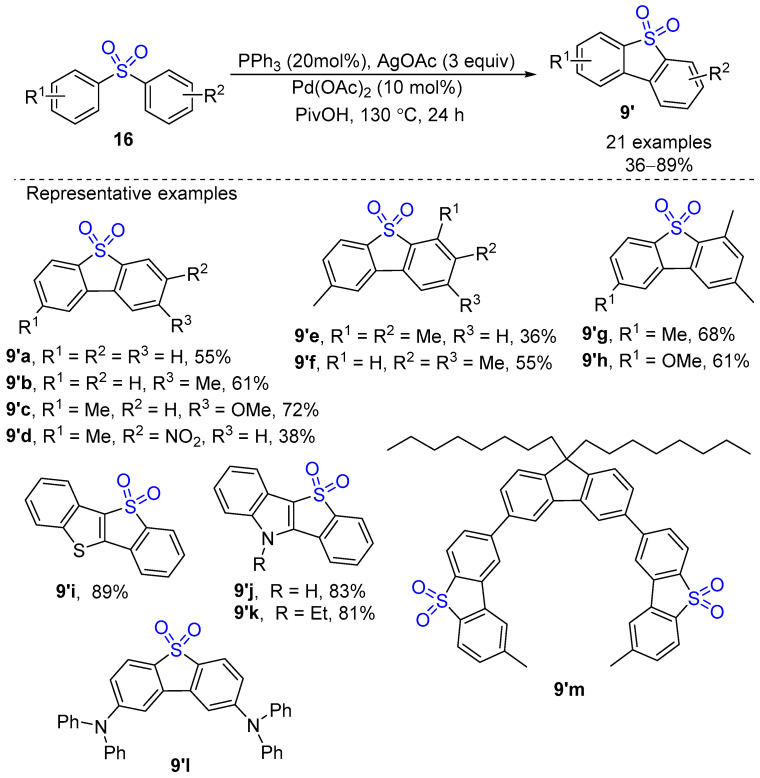
Palladium-catalyzed synthesis of fused biaryl sulfones.

In 2018, Laha and coworkers reported a novel palladium-catalyzed protocol to directly form fused biaryl and heterodiaryl sulfones **9′** through the intramolecular oxidative cyclization of two C(sp^2^) C−H bonds in biaryl sulfones **16** (Scheme 5) [33]. Variously substituted dibenzothiophene-5,5-dioxides **9′** could be smoothly prepared with yields ranging from 36% to 89%. It is worth mentioning that products **9′l** and **9′m** were effectively obtained using this reaction, as an α_7_-nicotinic acetylcholine receptor agonist analog and an organic emitter, respectively.

**Scheme 6:**
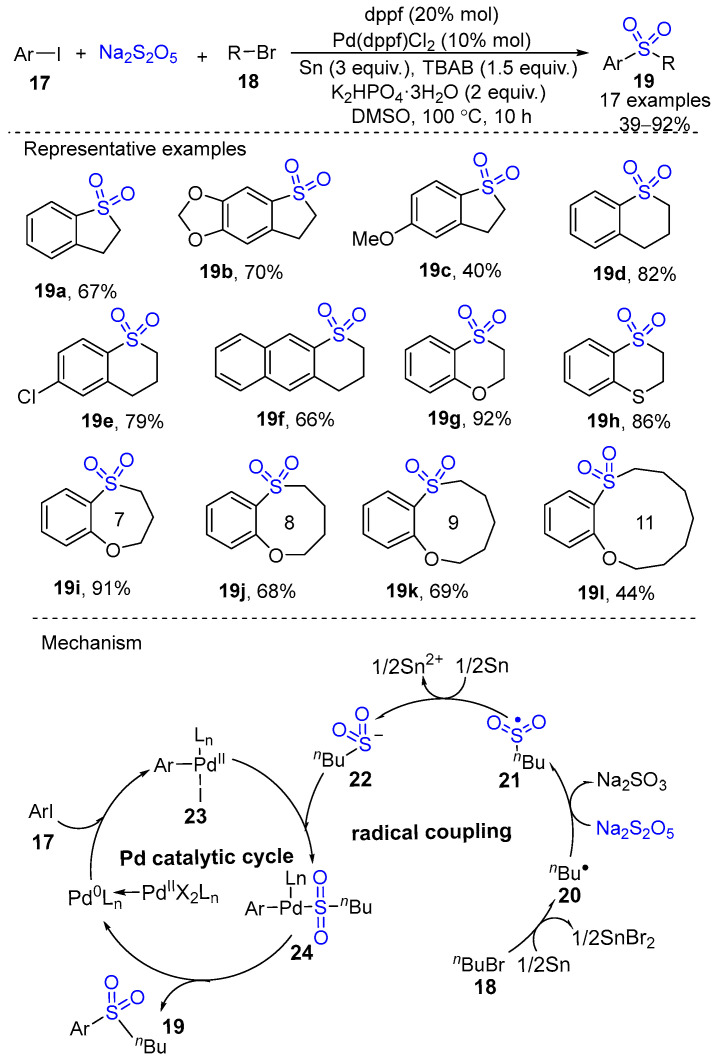
Palladium-catalyzed synthesis of five- to twelve-membered sulfones.

In 2020, Jiang et al. reported a novel multi-component strategy catalyzed by [1,1′- ferrocenebis(diphenylphosphino)] dichloro palladium(II) (Pd(dppf)Cl_2_), enabling the construction of five- to twelve-membered benzo-fused sulfones through a reductive cross-coupling reaction involving Na_2_S_2_O_5_, aryl halides **17**, and alkyl halides **18** (Scheme 6) [34]. The reaction was performed in DMSO at 100 °C using tin (Sn) as the reducing agent. A wide variety of electronically diverse aryl halides **17** were effectively transferred and gave the corresponding cyclic sulfones **19** in yields ranging from 39% to 92%, with smaller rings generally giving higher yields.

The proposed reaction mechanism involves the formation of a highly reactive alkyl radical intermediate **20** (^n^Bu**·**) through single-electron transfer between the *^n^*BuBr **18** and Sn. This intermediate **20** reacts with Na_2_S_2_O_5_ to generate sulfonyl radical **21**, which is reduced by Sn to produce sulfonyl anion **22**. Meanwhile, oxidative addition of aryl halide **17** to Pd⁰ forms intermediate **23**, which then reacts with sulfonyl anion **22**, yielding intermediate **24**. The desired cyclic sulfones **19** were obtained through reduction and elimination, with the Pd⁰ catalyst being regenerated in the process (Scheme 6).

**Scheme 7:**
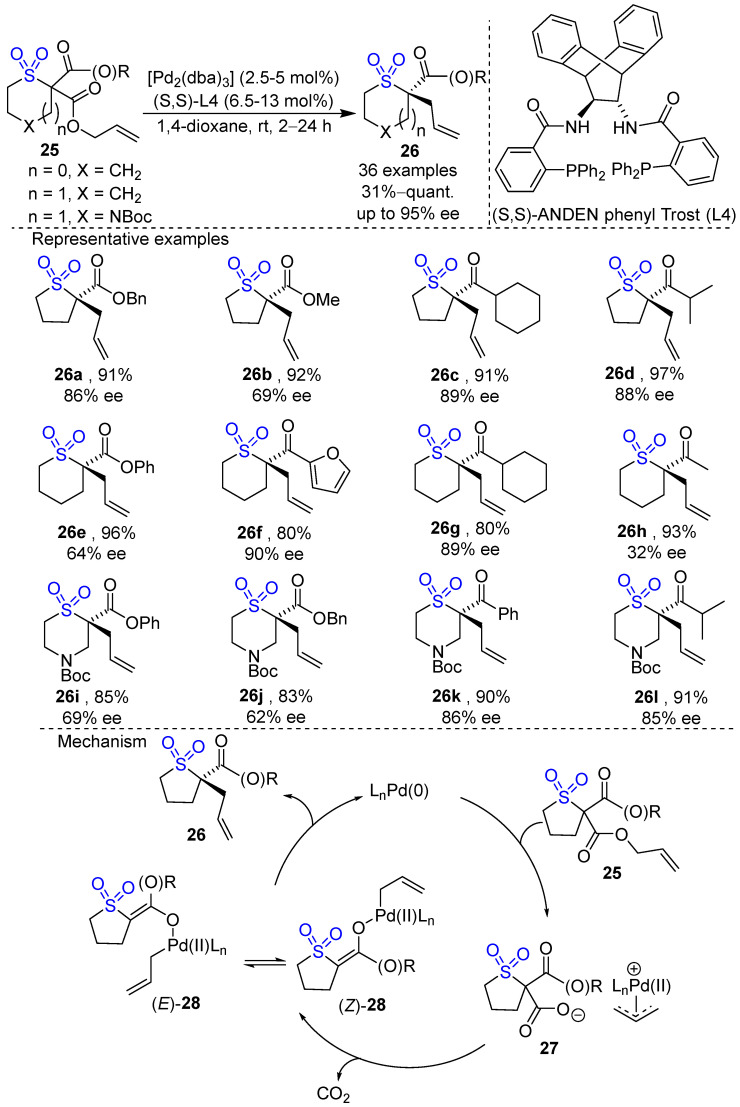
Palladium-catalyzed synthesis of *α*-difunctionalized 5- and 6-membered sulfones.

In 2022, Franckevičius and coworkers discovered a palladium-catalyzed decarboxylative asymmetric allylic alkylation (DAAA) reaction to afford enantioenriched *α*-difunctionalized cyclic sulfones **26** (Scheme 7) [9]. In this reaction, 5 mol% Tris(dibenzylideneacetone)dipalladium (0) [Pd_2_(dba)_3_] and 13 mol% (S,S)-ANDEN phenyl Trost ligand L4 were the best catalyst choices. A range of racemic ester- and ketone-substituted sulfone substrates **25** were investigated, and the relevant expected products **26** were isolated in moderate to excellent yields with high stereoselectivity. The innovation of this approach lies in achieving high levels of enantioselectivity through the dynamic kinetic resolution of *E-* and *Z-*enolate intermediates.

The reaction was initiated by oxidative addition of the Pd (0) catalyst to allyl ester **25**. The resulting intermediate **27** is a loosely associated ion pair between a carboxylate and a π–allylpalladium(II) complex, which undergoes rapid CO_2_ extrusion to give a mixture of *E-* and *Z-*enolates **28**. Rapid isomerization occurs between (*E*)-**28** and (*Z*)-**28**, presumably through a carbon-bound palladium enolate tautomer, followed by preferential allylic alkylation of (*Z*)-**28** over (*E*)-**28**, leading to the formation of the enantioenriched product **26**.

## 3. Photocatalytic Synthesis of Cyclic Sulfones

Nowadays, visible light-induced organic synthesis has gained considerable research attention and is considered as a green, sustainable, and cost-effective synthetic methodology for the preparation of diverse carbocyclic and heterocyclic compounds [35,36].

**Scheme 8:**
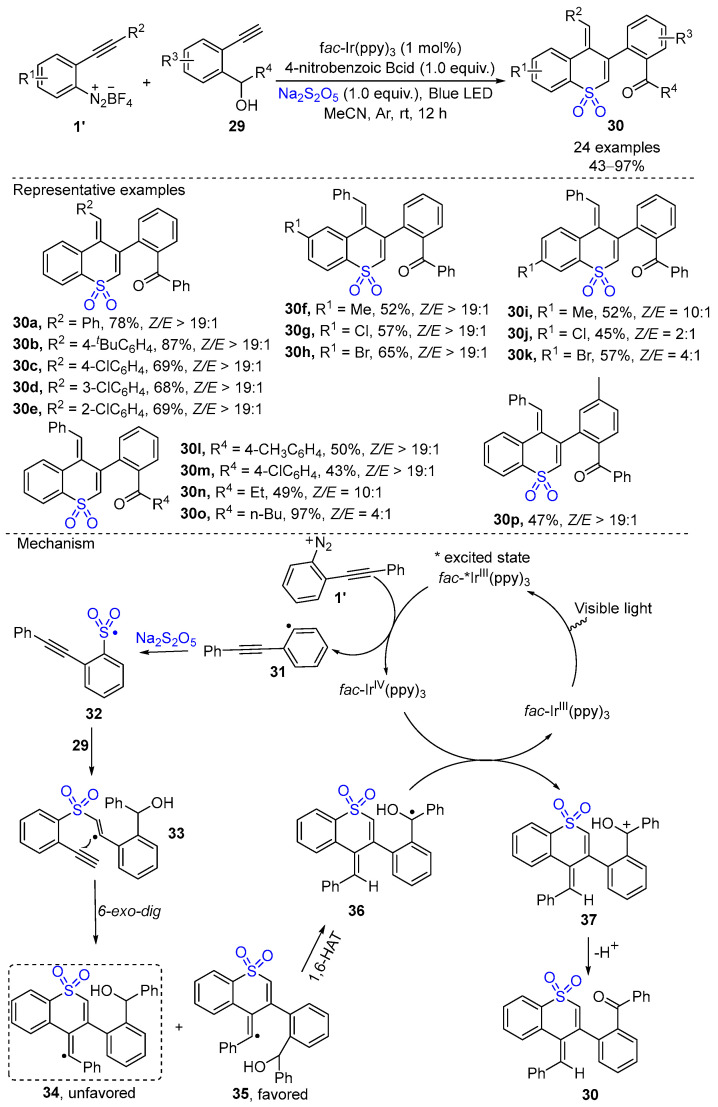
Photocatalytic synthesis of dicyclic sulfones.

In 2021, Jiang and colleagues reported a novel photocatalytic three-component cycloaddition cascade reaction of 2-alkynylaryldiazonium tetrafluoroborates **1′** with γ-hydroxyl terminal alkynes **29** (Scheme 8) [37]. This transformation employed *fac*-tris(2-phenylpyridine)iridium (*fac*-Ir(ppy)_3_) as the photocatalyst [38] and 4-nitrobenzoic acid as an additive under blue light irradiation; functionalized bicyclic sulfones **30** were obtained in good yields with high stereoselectivity (*Z*). The experimental results indicated that this photocatalytic cyclization was highly effective for *γ-*hydroxy terminal alkynes **29** bound to phenyl rings. Moreover, altering the substituents on acetylene motifs of 2-alkynylaryldiazonium tetrafluoroborates **1′** had minimal impact on reaction activity. Substrates **29** bearing Me, Cl, and Br at the C5 position of the aromatic ring were readily converted to the corresponding (*Z*)-products **30i–30k** with acceptable yields and stereoselectivities (2:1 to 10:1 *Z/E* ratio). Further investigations showed that the aryl groups on secondary alcohols of **29** could be replaced with ethyl and *n*-butyl groups, thus expanding the scope of the photocatalytic reaction.

The proposed mechanism shows that a single-electron transfer (SET) process occurs between aryl diazotetrafluoroborate **1′** and the excited *Ir(III) complex, generating an aryl radical intermediate **31** and Ir(IV) species simultaneously. The aryl radical **31** then undergoes a reaction with Na_2_S_2_O_5_, yielding an aryl sulfonyl radical **32**. Radical **32** undergoes intermolecular addition, followed by a *6-exo-dig* cyclization with *γ*-hydroxy terminal alkyne **29**, forming the exocyclic vinyl radical **35** as the predominant isomer. Due to steric hindrance from aryl and hydroxymethyl groups, intermediate **34** is less favorable for hydrogen abstraction. Intermediate **35** undergoes a 1,6-HAT (hydrogen atom transfer) process, resulting in hydroxymethyl radical **36**. This radical is subsequently oxidized by Ir(IV) to furnish the hydroxymethyl cation **37**, which regenerates the Ir(III) complex. Finally, intermediate **37** undergoes deprotonation to yield the desired (*Z*)-thiochromene 1,1-dioxides **30** (Scheme 8).

**Scheme 9:**
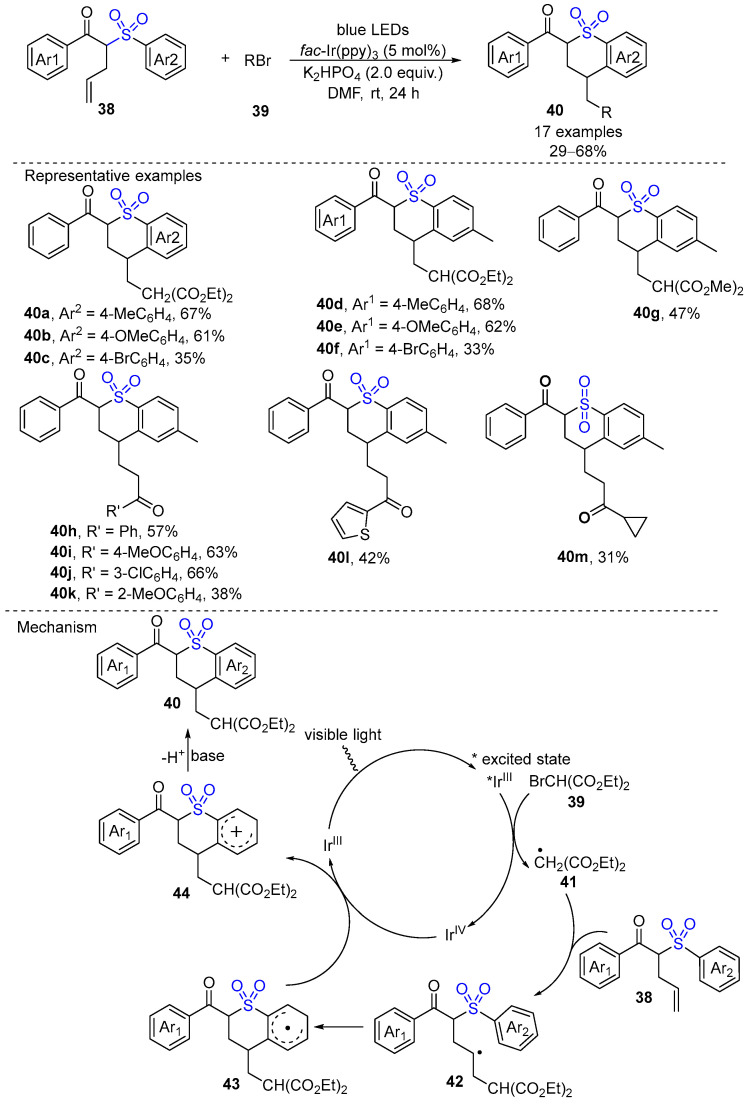
Photocatalytic synthesis of 2-benzoyl-1,1-dioxidothiochroman-4-yl derivatives.

In 2023, Huang’s research group reported an alkyl radical-initiated difunctionalization reaction of non-activated alkanes to synthesize the 2-benzoyl-1,1-dioxidothiochroman-4-yl derivatives **40** (Scheme 9) [39]. This reaction utilized *α*-carbon-based alkyl bromide **39** as the oxidant and 450 nm LEDs as the light source; *α*-allyl-*β*-ketosulfones **38** bearing various substituents could be transformed into the corresponding sulfones **40** in 29–68% yields, revealing that electron-donating groups favored the reaction. Additionally, different oxidants, such as *α*-bromoalkyl ester, 2-bromoacetophenones, and 2-(bromoacetyl)thiophene, also worked well.

The proposed mechanism is illustrated in Scheme 8. The photoexcited Ir(III)* catalyst is oxidized sufficiently to carry out SET with *α*-carbon-based alkyl bromide **39** forming Ir(IV) and alkyl radical **41**. Radical **41** then adds to substrate **38,** thereby producing alkyl radical **42**. Radical **42** subsequently undergoes an intramolecular radical cyclization, resulting in the generation of the stable intermediate **43**. The photocatalyst Ir^IV^ accepts an electron from intermediate **43** to afford cationic intermediate **44**, which then delivers the final product **40** after deprotonation.

## 4. Other Methods for Synthesizing Cyclic Sulfones

Recently, as research continues to advance, researchers have developed a variety of metal-free and efficient methods for synthesizing cyclic sulfone compounds with complex structures and diverse functionalities [40]. By eliminating the use of metal catalysts, metal-free strategies reduce costs and environmental impact.

**Scheme 10:**
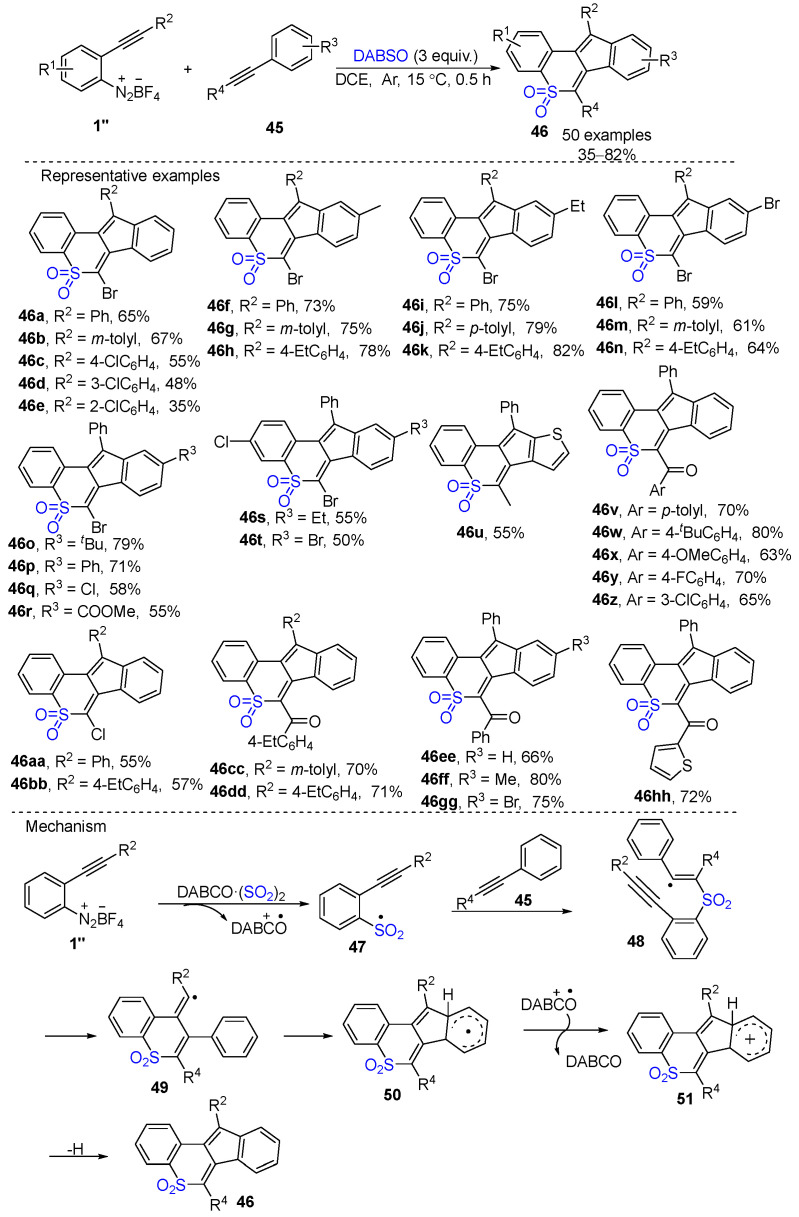
The synthesis of polycyclic sulfones.

In 2019, Jiang and coworkers developed a radical-initiated, three-component double-ring cascade reaction mediated by DABSO (Scheme 10) [41]. Under mild and neutral redox conditions, the expected products, indeno [1,2-*c*]thiochromene 5,5-dioxide **46**, could be obtained in 35–82% yields, with tolerance of various functional groups, including aryl, alkyl, and heterocyclic groups.

A radical mechanism was verified by 2,2,6,6-tetramethyl-1-piperidinyloxy (TEMPO) control experiments. The combination of DABSO and substrates **1″** generates the arylsulfonyl radical **47** and a tertiary amine (DABCO) radical cation. Radical **47** then adds to the triple bond of haloalkynes **45**, providing the alkenyl radical intermediate **48**. Intermediate **50** arises from intermediate **49** through further *6-exo-dig* cyclization/*5-endo-trig* cyclization, which loses an electron to access intermediate **51**. Subsequent deprotonation gives rise to the formation of product **46**.

**Scheme 11:**
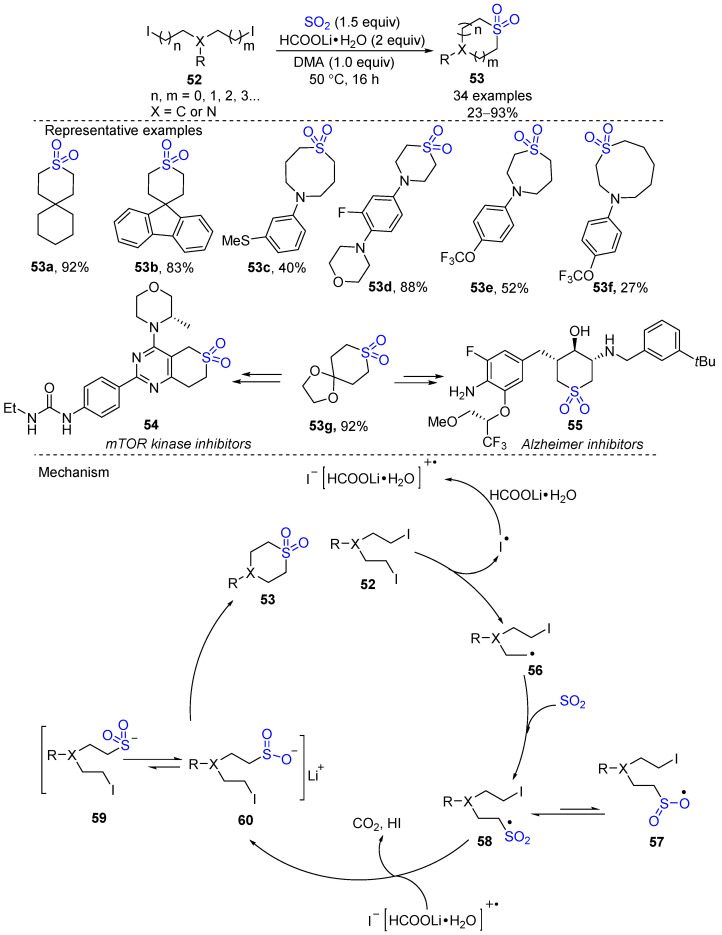
The synthesis of cycloaliphatic sulfones.

In 2021, Lian and colleagues successfully established a method for synthesizing cyclic aliphatic sulfones through the sulfonyl cyclization of alkyl diiodides **52** and gaseous SO_2_ (Scheme 11) [42]. The reaction of N,N-bis(2-iodoethyl)aniline **52**, HCOOLi·H_2_O and SO_2_ in dimethylacetamide (DMA) at 50 °C for 16 h led to the formation of nitrogen-containing five- to nine-membered cyclic aliphatic sulfones **53**. The substrate scope demonstrated the practicality of this method, as a variety of alkyl diiodides yielded moderate to high yields of cyclic sulfones with good efficiency. Notably, product **53g** could be transformed to mTOR kinase inhibitors **54** and Alzheimer’s inhibitors **55** via multi-step synthesis.

The proposed mechanism involves the cleavage of the C-I bond in alkyl diiodide **52** to form alkyl radical intermediate **56**. This radical then reacts with SO_2_ to generate alkyl sulfonyl radical intermediate **57**, which is in equilibrium with another radical intermediate **58**. Subsequently, intermediate **58** is reduced by a formate radical cation, resulting in the formation of alkyl sulfonyl anion **59**. This anion may exist in equilibrium with alkyl sulfonate **60**, which undergoes intramolecular nucleophilic substitution to yield the desired cyclic sulfone.

**Scheme 12:**
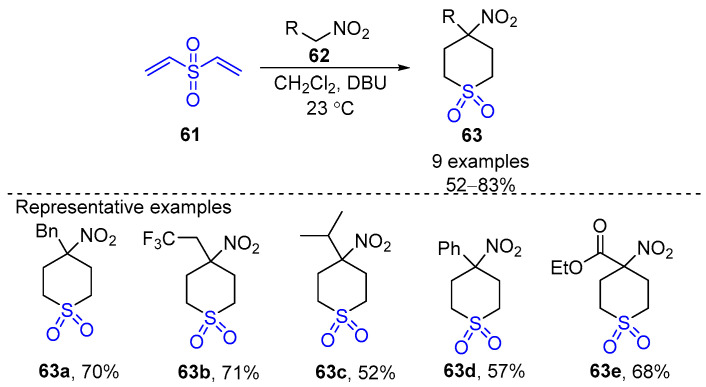
The synthesis of 4,4-disubstituted nitro sulfones.

In 2021, Hugelshofer and Bao reported a Michael reaction to form various 4,4-disubstituted nitro sulfones **63** using divinyl sulfone **61** and commercially available raw materials **62**. Substituted alkyl nitro, benzyl nitro, and *β*-nitro ester derivatives, acting as active methylene nucleophiles, were smoothly introduced to a wide range of cyclic sulfones (Scheme 12) [43].

**Scheme 13:**
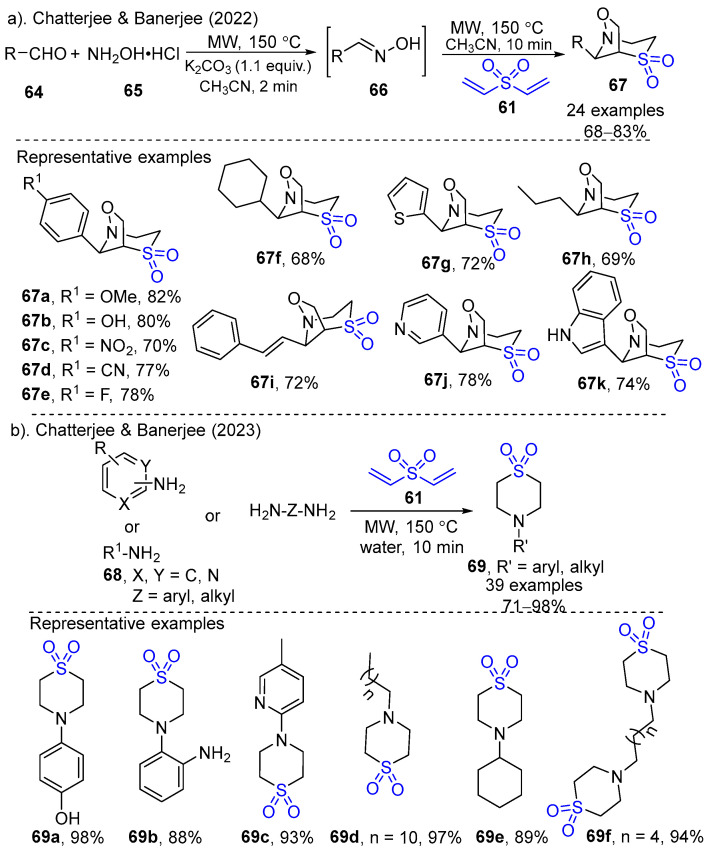
Microwave-assisted synthesis of cyclic sulfones (**a**) [45] (**b**) [46].

In addition, a sustainable, catalyst-free microwave method [44] has quickly gained prominence, which involves in situ aldoxime formation followed by a tandem *aza*-Michael addition-1,3-dipolar cycloaddition. This reaction system utilizes microwave radiation as an energy source, significantly reducing reaction time while eliminating the need for expensive catalysts, fully embodying the principles of green chemistry. Aldehyde **64**, hydroxylamine hydrochloride **65**, and potassium carbonate (K_2_CO_3_) were added to a microwave reactor, and the mixture was heated at 150 °C for 2 min to yield the corresponding aldoximes **66**. These aldoximes **66** then reacted with divinyl sulfone **61** in the same pot to gain the desired product **67** (Scheme 13a) [45]. Aromatic, heteroaromatic, and aliphatic aldehydes were suitable substrates. The reaction could be carried out on a gram scale with high efficiency, offering facile access to bicyclo *aza*-sulfones. One year later, Banerjee and Chatterjee applied the microwave catalytic system to the double *aza*-Michael addition reaction (Scheme 13b) [46]. This reaction proceeds on the water surface under microwave irradiation at 150 °C for 10 min, where amines **68** and divinyl sulfone **61** were transformed into solid cyclic *β*-amino sulfones **69** with excellent yields. Both electron-rich and electron-deficient amines participated effectively in this transformation, demonstrating good functional group tolerance. In particular, the method is sustainable due to its reagent-free, metal-free, organic solvent-free, and 100% atom-economic nature.

**Scheme 14:**
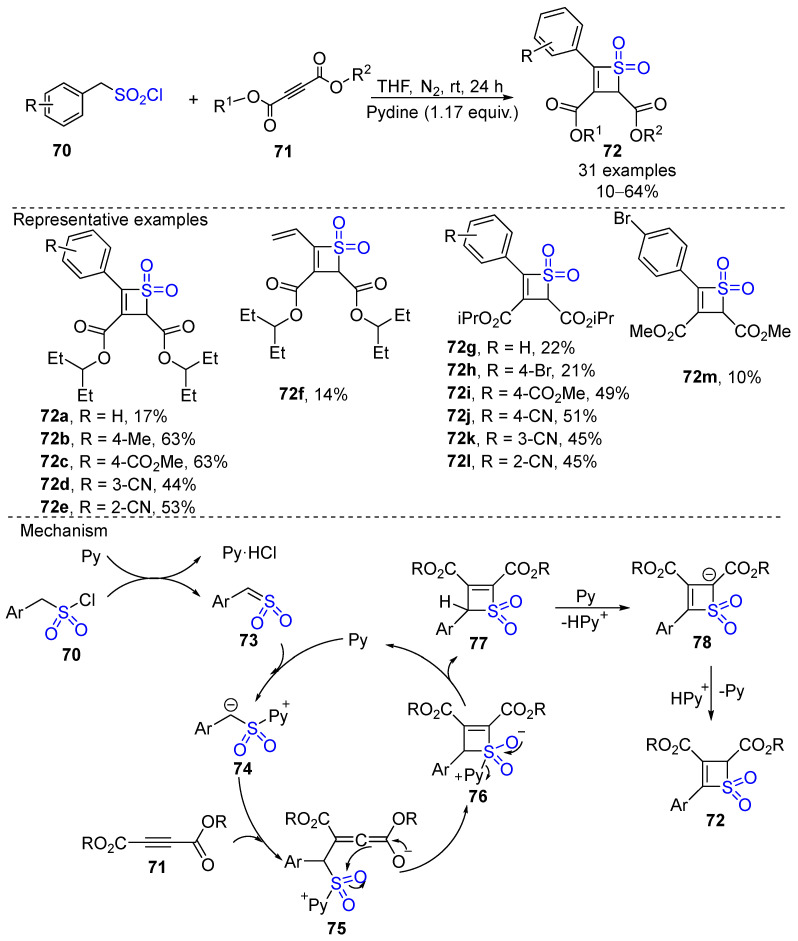
The synthesis of bifunctionalized 2*H*-thiete 1,1-dioxide derivatives.

In 2022, Xu and coworkers developed a pyridine-mediated [2 + 2] cycloaddition reaction to synthesize bifunctionalized 2*H*-thiete 1,1-dioxide derivatives **72** from readily available sulfonyl chloride **70** and dialkyl acetyl dicarboxylic acid **71** (Scheme 14) [47]. The reaction was conducted by stirring sulfonyl chloride **70**, acetoacetyl dicarboxylic acid **71**, pyridine, and tetrahydrofuran (THF) at room temperature under a nitrogen atmosphere for 24 h to yield the desired products **72**. Various sulfonyl chlorides were tested, including di(pentan-3-yl) acetylenedicarboxylate. The results indicated that benzene sulfonyl chloride with electron-donating groups did not undergo the annulation. To assess the impact of steric hindrance in dialkyl acetyl dicarboxylates **71**, diisopropyl acetyl dicarboxylate was tested with different electron-deficient benzyl sulfonyl chlorides. The findings revealed that stronger electron-withdrawing sulfonyl chlorides produced higher yields than their weaker counterparts. Additionally, for the same sulfonyl chloride, di(pentan-3-yl) esters yielded better results compared to the corresponding diisopropyl esters, highlighting the significant effect of steric hindrance on the reaction yield.

A proposed mechanism involves the generation of sulfonic acid **73** from sulfonyl chloride in the presence of basic pyridine. Following the elimination of hydrochloric acid, pyridine acts as a nucleophile, attacking the sulfonyl group of sulfonic acid **73** to form zwitterionic intermediate **74**. This intermediate then nucleophilically attacks dialkyl acetyldicarboxylic acid **71**, contributing to the formation of another zwitterionic intermediate **75**. The enoate of intermediate **75** is eliminated through intramolecular nucleophilic addition to sulfonyl pyridine, generating intermediate **76**. Subsequently, intermediate **78** is isomerized via intermediate **77** to produce the final four-membered thiaheterocyclic derivatives **72**.

**Scheme 15:**
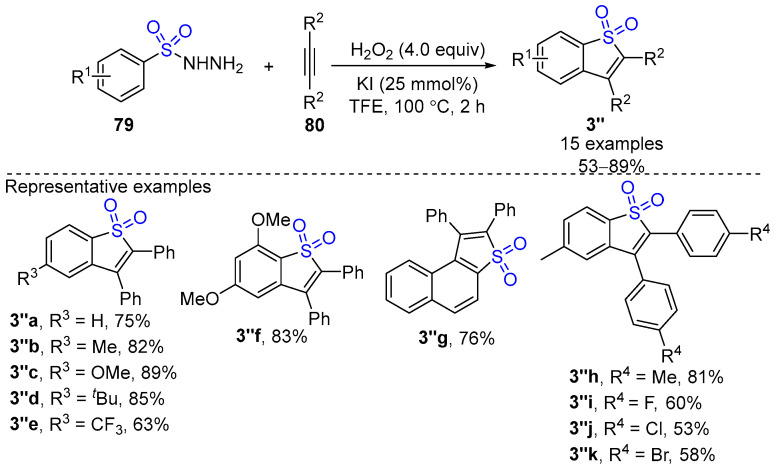
The synthesis of benzo[*b*]thiophene-1,1-dioxides.

Remarkably, in 2019, Sun and coworkers reported a solvent-controlled strategy to obtain benzo[*b*]thiophene-1,1-dioxides **3″** via a radical pathway (Scheme 15) [48]. The reaction was performed in 2,2,2-trifluoroethanol (TFE), rapidly occurring in the presence of simple hydrogen peroxide and potassium iodide (KI), without the need for any transition metal catalysts. The use of *para*-toluenesulfonyl hydrazone with a strong electron-donating methoxy group facilitated the controlled radical reaction, resulting in exceptionally high yields of 89% for product **3″c**. It should be noted that product **3″d** presents aggregation-induced emission (AIE) properties.

**Scheme 16:**
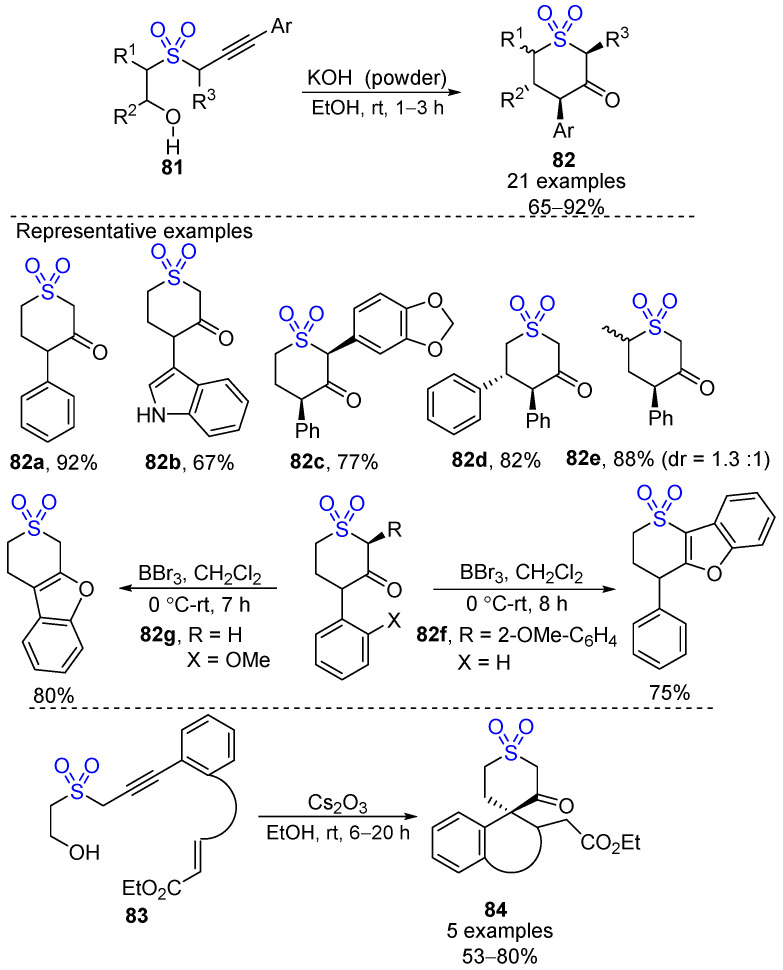
Base-promoted synthesis of cyclic *β*-ketosulfones.

In recent years, base-promoted organic reactions have made significant advancements in the construction of C–S [49] or C–C [50] bonds. In 2023, Gharpure and coworkers reported an efficient method for the stereoselective synthesis of cyclic *β*-ketosulfones **82** using various sulfone-tethered alkynols **81** promoted by KOH (Scheme 16) [7]. This cascade transformation proceeded through a base-driven propargyl sulfone undergoing allene isomerization/intramolecular/hydroalkoxylation/retro-*oxa*-Michael/a *6-endo-trig* Michael addition. Products **82f** and **82g** can be readily converted into the corresponding benzofuran-fused cyclic sulfones in 75% and 80% yields, respectively. With Cs_2_CO_3_ as the base, sulfone-tethered alkynyl acrylates **83** were transformed into spirocyclic *β*-ketosulfone **84** with yields ranging from 53% to 80%. This work features excellent substrate adaptability and synthetic applicability, paving the way for the green synthesis of more complex molecules.

## 5. Conclusions and Outlook

In summary, we have reviewed the latest advancements in the efficient synthesis of cyclic sulfones by catalytic protocols. Transition metal-catalyzed synthesis of cyclic sulfones highlights the regioselectivity and high yield across a diverse range of substrates; however, the reliance on specific catalysts, such as Pd(dppf)Cl_2_ and Pd_2_(dba)_3_, may impose challenges in terms of scalability and cost. Furthermore, the reaction conditions may require careful optimization to accommodate a range of functional groups. Visible light-driven synthesis of cyclic sulfones aligns with the principles of green chemistry, making the process more sustainable. These reactions are conducted at room temperature, reducing the need for harsh chemicals or extreme conditions that could potentially degrade sensitive substrates. Photocatalytic methods, although promising, face challenges when scaling from the laboratory to industrial scale. Issues such as the cost of photocatalysts, light absorption efficiency, and reaction optimization for large-scale processes must be addressed.

Nevertheless, there is an ongoing high demand for novel, versatile, and sustainable methods for synthesizing structurally diverse cyclic sulfones and their applications in the construction of bioactive compounds and other useful derivatives. Meanwhile, experimental mechanistic studies will be crucial for gaining a deeper understanding of the reaction details. We anticipate that more groundbreaking and impactful work in this field will emerge in the near future.

## Data Availability

Not applicable.
